# Design and Evaluation of Flooding-Based Location Service in Vehicular Ad Hoc Networks†

**DOI:** 10.3390/s20082389

**Published:** 2020-04-22

**Authors:** Paul Mühlethaler, Éric Renault, Selma Boumerdassi

**Affiliations:** 1 EVA Project, Inria - Paris, 75012 Paris, France; 2 LIGM, University Gustave Eiffel, CNRS, ESIEE Paris, 93160 Marne-la-Vallée, France; Eric.Renault@telecom-sudparis.eU; 3 CEDRIC/ CNAM, 75003 Paris, France; selma.boumerdassi@cnam.fr

**Keywords:** Location Services, VANETs, Flooding, Semiflooding, MPR Flooding, Scaling, Analytical Model

## Abstract

Location-based routing protocols for vehicular ad hoc networks (VANETs) use location information to determine routing decisions. This information is provided by a location service that is queried by nodes in order to properly forward packets to communication partners. This paper presents the semiflooding location service, a proactive flooding-based location service that drastically reduces the number of update packets sent over the network compared to traditional flooding-based location services. This goal is achieved by each node partially forwarding location information. We present both deterministic and probabilistic approaches for this algorithm, which remains very simple. A mathematical model is proposed to show the effectiveness of this solution. The cases of homogeneous 1D, 2D, and 3D networks were studied for both deterministic and probabilistic forwarding decisions. We compare our algorithm with simple flooding and with the multipoint-relay (MPR) flooding of the optimized-link-state-routing (OLSR) protocol, and we show that our algorithm, despite being very simple, has excellent scalability properties. The mean number of generated messages ranges with the mean number of the neighbors of one random network node.

## 1. Introduction

Research in the field of intelligent transport systems (ITSs) [1] is very active. Numerous advances have been made to increase user safety and comfort. Vehicular ad hoc networks (VANETs) are part of ITS, and developing them to obtain completely autonomous vehicles is a necessity. One of the most important characteristics of VANETs is their dynamic topology [2], which is a consequence of the network being made up of mobile nodes: nodes can change their position quite frequently. This means that there is a need for routing protocols that are capable of quickly adapting to changes in network topology. The geographical location of nodes (i.e., node co-ordinates) has been suggested as a means to address this issue. Location-based (geographic) routing protocols base their routing decisions on location information using the location of the destination node to forward packets to this node [3,4,5]. Establishing the destination’s location is the first step towards communicating with the destination. Different means are available for a node to identify its own position, and the GPS is certainly the best-known and most used [6,7]. Determining its own position is usually locally done by the node, even though other solutions exist. However, identifying the location of the destination node when sending a message cannot be done locally without the use of external data or services. In geographic routing protocols, this is achieved by means of a location service. This service is used by the sender of a packet to determine the location of the destination. As a result, the performance of a geographic routing protocol mainly depends on the performance of the location service.

**Figure 1:**
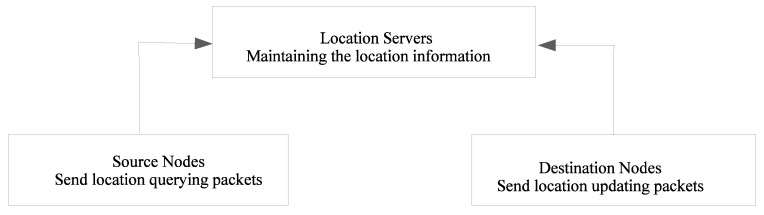
General architecture for a location service.

A location service provides the location information for a specific node in a VANET by implementing a mechanism that tracks the location of nodes in the network. Figure 1 shows the general structure of a location-service management protocol. The scheme essentially consists of three different entities (a location service, a source node, and a destination node), and two main operations (a location query and a location update). Every node updates the location service responsible for handling the location information. When a node needs to send a packet, it queries the location service and retrieves the destination’s location. The location service of a node may be situated on the source node itself or on another node. When the location service is present on the source node, the query is locally resolved by the source node, as is the case for location-information dissemination methods.

A location-service protocol has to exhibit the following properties:

- Efficiency: induced overhead by control packets (e.g., location updates and location queries) should be kept to a minimum.
- Robustness: the location service should not be disrupted by node mobility or failure (especially for location servers if they are separate nodes), or disconnection from the network.
- Load balancing: the protocol should apply traffic-balancing schemes to avoid bottleneck problems.
- Scalability: the protocol should maintain all of the above properties when the network scales to a large number of nodes.
- Locality awareness: the distance of the location server from the querying source should not be greater than the distance between the source and the destination nodes. In other words, location queries for nearby nodes should not travel unnecessarily over the network. However, the benefits of this feature rely on the presence of local data-traffic patterns.

This article provides the following contributions:

- A new location-service protocol that limits the amount of control information exchanged between nodes while maintaining accurate location information. Two versions of the protocol are proposed, a deterministic and a probabilistic version.
- An analytical model to evaluate the number of messages sent by the protocol is provided. Models were developed for 1D, 2D, and 3D networks.
- Simulations to confirm the validity of the analytical model are presented.
- Comparisons with the simple flooding technique and the optimized-link-state-routing (OLSR) multipoint-relay (MPR) [8] flooding technique are provided, which show the scaling properties of our algorithm.

The algorithm presented in this paper (semiflooding location service, SFLS) was previously published in the Mobile and Wireless Networking Symposium of the IEEE ICC 2016 Conference [9]. However, the present paper introduces new material: a probabilistic version of the algorithm, a better analytical model with both the probabilistic algorithm and the 3D network case, simulation results, and scaling analysis that compares our algorithm with simple flooding and with an improved flooding scheme, i.e., the MPR flooding of OLSR.

## 2. Related Work

Location services for VANETs can be divided into two categories. In the first, protocols use information from the network of vehicles and from the urban infrastructure, such as cellular-network base stations or road-side units (RSUs) [10]. This infrastructure is usually present on the roads. This allows reliable positions to be obtained by reducing the number of signaling messages exchanged between nodes. However, these algorithms are dependent on urban-structure providers and do not allow a spontaneous VANET to emerge. The second category of algorithms does not rely on any urban infrastructure, and thus allows the creation of independent spontaneous networks. Our proposal falls within the second kind of location services.

The state of the art shows that all proposals for this second kind are based on the choice of the number of vehicles that are used as location servers (usually called rendezvous vehicles) [11]. These elected nodes store and distribute location information. The performance of such algorithms also primarily depends on the choices made to elect those servers. In VANETs, another way to set up a location service consists of sharing location information between all nodes in the network using broadcast messages. The main advantage of this technique is that it eliminates the phase of choosing the servers, this phase often being quite complex. Its main drawback is that flooding may overload the network, which can lead to the rapid degradation of overall performance.

In this article, we propose a flooding-based mechanism for VANETs where a partial broadcast of location-information packets is performed. The following are the most notable location algorithms found in the literature.

### 2.1. Region-Based Location-Service Management Protocol (RLSMP)

The RLSMP [12] is a rendezvous-based location service that divides the network into regions (called segments) that are further divided into cells. Each cell has a cluster leader (CL) that manages the location of all nodes in the cell, and sends its location information to the cell responsible for the cluster, called the location-service cell (LSC). A vehicle querying for destination co-ordinates sends its location query to its LSC. If the requested information is not found in this LSC, the query is forwarded to neighboring LSCs, following a spiral shape until the co-ordinates of the destination are found. The RLSMP generates a significant signaling load, as the same query may visit several LSCs before reaching the one holding the requested information. This approach provides reliable location information. However, a lot of traffic must be generated to keep the cluster leaders up to date.

### 2.2. Vehicle-Location Service (VLS)

The VLS [13] is a rendezvous-based location service that uses information from digital maps to partition the network and avoid the creation of empty zones (i.e., zones with no active nodes). Each zone contains a location server. The location server of a vehicle is obtained using the vehicle identifier and a hash function. Hence, a vehicle can send its location-update message using the co-ordinates of the nearest location server. Location updates are sent to the nearest location server, and location queries are sent to the nearest location server of the queried vehicle. When leaving its zone, each server has to transfer its location information to a new server. In high-mobility configurations, the VLS protocol induces a high overhead.

### 2.3. Density-Aware-Map-Based Location Service (DMBLS)

The DMBLS [14] is a location service protocol where the network zone is divided into a hierarchy of regions. A region is a network zone with high traffic density, and all the location servers are chosen in this region. In this protocol, queries from vehicles are forwarded to the higher levels until the required information is found and sent back to the client. The performance of this protocol is highly dependent on the choice of region, especially when considering the changing characteristics of traffic during different periods of the day.

### 2.4. Zoom-Out Geographic-Location Service (ZGLS)

The ZGLS [15] is a quorum-based location service. A quorum is a subset of nodes in the network [16]. In the scope of the ZGLS, each node is associated with two quora, the update quorum that is used to update the location information, and the query quorum that is used to look for the location of another node. For a single node, these two quora can be different. However, they are designed so that the intersection of the query quorum of each node with the update quorum of any of the other nodes is not empty. This ensures that the update quorum always satisfies the query. The specificity of ZGLS, according to the authors, is that location updates and answers are done through single-hop communications. In order to provide quora for each node, a one-hop location server called the parent is found. All parents are included in a chain of parents. This leads to a very good response time for location queries at the expense of a very high cost for setting up the infrastructure, especially regarding the identification of all quora.

### 2.5. Vehicle-Aided Location Service (VALS)

The VALS [17] is a location service in urban environments. It is based on the use of a hierarchy of location servers. VALS forms clusters of vehicles. Each cluster chooses a cluster head, whose role is to supply the location servers with the current position of its members. In VALS, an urban area is divided into two levels of hierarchies. The first level consists of road-side units (RSUs) located at each intersection of the area. The cluster head regularly sends its location to the nearest RSU. At Level 2 of the hierarchy, regional location servers (RLS) are used to regularly collect the location tables of the nearest RLS. This service can only operate in urban areas.

### 2.6. Mobile-Group-Based Location Service (MoGLS)

The MoGLS [10] uses a hierarchical structure of location servers. The lower level is made up of dynamic groups of vehicles with similar trajectories. Each group has a cluster head that acts as the location server for its group. The upper level is made up of fixed servers. Cluster heads regularly send their location base to fixed servers. MoGLS offers the data aggregation and dynamic management of vehicle groups. This location service can only operate with a fixed infrastructure, and the management of the group of vehicles can quickly generate significant traffic overhead, particularly in the event of high density and mobility.

## 3. Semiflooding Location Service

The semiflooding location service (SFLS) was developed to take advantage of both flooding- and rendezvous-based location services for VANETs while avoiding their drawbacks. The advantage of flooding-based solutions is that each node in the network is aware of the position of all the other nodes, while the advantage of rendezvous-based solutions is the low number of messages exchanged to keep the positions of the nodes up to date. The drawback of flooding solutions is the high number of messages exchanged between nodes (location messages are forwarded without any change to the boundary of the network), while the drawback of rendezvous solutions is their difficulty to handle node disappearance and communication problems. The SFLS allows all nodes in the network to have accurate-enough location information about all other nodes in the network, while not overwhelming the network with location packets. Moreover, SFLS is resilient to node disappearances and network problems.

### 3.1. Principle

The main idea of the semiflooding location service is as follows. All nodes in the network periodically broadcast a packet containing their location information with the time when the location was issued. Nodes do not need to be synchronized for this operation, i.e., all nodes’ internal times do not need to be aligned to a single time reference, and location packets can even be broadcast at different updating rates if needed. When a node receives a location packet, it first compares the timestamp in the location packet with the one stored in the node’s location table. If the incoming information is older than or equal to the locally stored one, the incoming packet is discarded. Otherwise, the new location information is saved, replacing the former information, and 1 in *q*, the packet is forwarded to the neighbors. The way parameter 1/q is used is twofold. On the one hand, 1/q can be seen as a probability, in which case a random generator can be used to determine whether the packet has to be forwarded. If the value returned by the (pseudo)random generator is smaller than 1/q, the packet is forwarded; otherwise, it is not. On the other hand, 1/q can be understood as a threshold for deterministic forwarding. For example, if q=3, only one packet in three is forwarded, i.e., the first two are not forwarded, while the last one is. The value for *q* is not necessarily an integer and could be any real number greater than 1. The closer to 1, the higher the frequency of packet forwarding.

**Figure 2:**
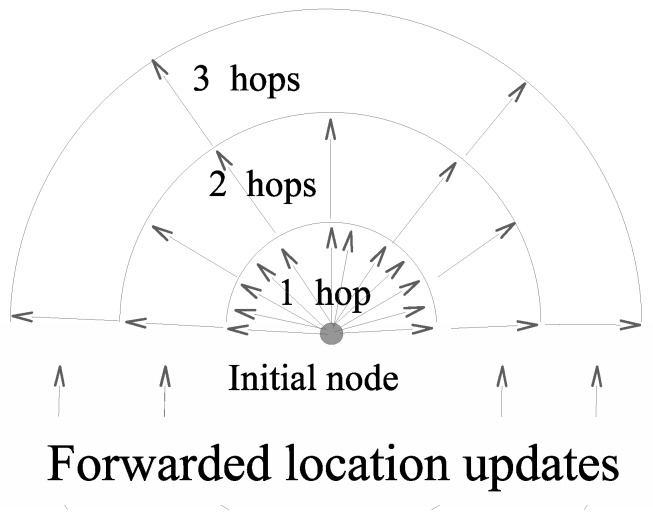
Semiflooding location service.

Figure 2 shows how far location packets are propagated at each location update when *q* is equal to 2, and a deterministic algorithm is used. In this example, the longest path from the node emitting the location packets to any other nodes is 3. The first location packet (the first arrow clockwise in Figure 2) reaches all nodes in the network, i.e., those that are at a distance of up to three hops from the emitting node. The second location packet (the second arrow in the same direction) only reaches one-hop nodes. The third location packet (and the same goes for packet numbers 7, 11, 15, etc.) reaches nodes at one and two hop(s). The fourth location packet (and any other odd-ranked location packet) only reaches nodes at one hop. Location packet 5 reaches all nodes up to three hops away.

Figure 2 also highlights the fact that the greater the distance from the node emitting the location update is, the longer the period of time between two location updates. However, for most applications (including routing), the greater the distance between two nodes is, the less accurate the knowledge of the other node’s position is. Most of the time, an approximate position is more than enough, the accurate location of the destination node becoming increasingly important as a data packet gets closer to the destination node. As a result, only significant changes in node locations should be reported, and this is exactly what the semiflooding location service does.

### 3.2. Algorithms

**Figure 3:**
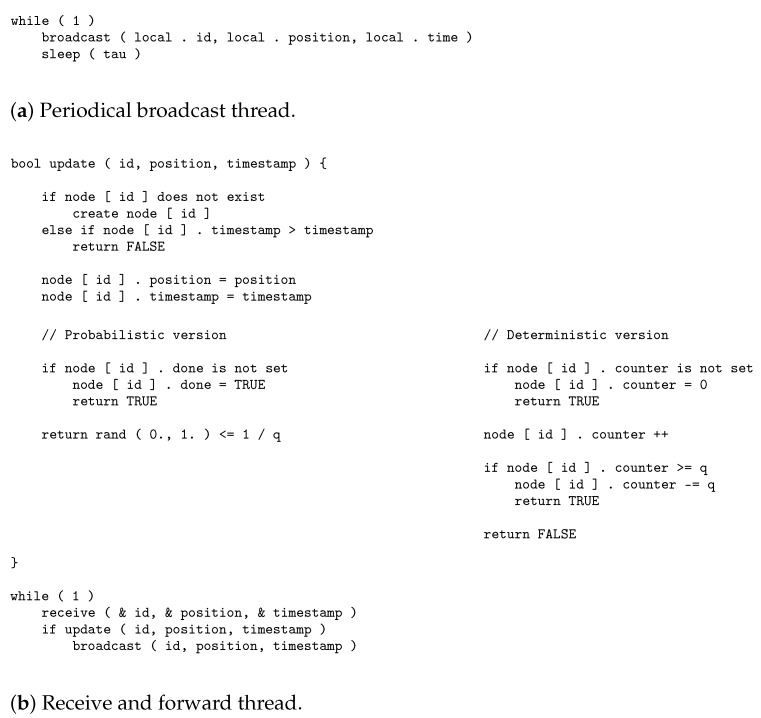
Semiflooding-location-service (SFLS) algorithms.

The semiflooding location service can be formalized using two algorithms running asynchronously on all nodes in the network. The first algorithm (see Figure 3a) aims at periodically broadcasting local location information. The second (see Figure 3b) aims at updating the local location information of other nodes when an update packet that is received and forwarding it 1 in *q*.

Each node in the network stores an array (node) to save information about the other nodes. This array is indexed by the node ID. Information for each node includes:

- position, the most recent position issued by the node and received locally.
- timestamp, the time the position was issued. This timestamp is the one given by the issuer node and not by the receiver. Synchronization is not an issue since only timestamps related to the same node are compared.
- done, a Boolean value that can be used to make sure that, the first time a location position is issued for a node, this message is forwarded up to the boundary of the network. This property is only available for the probabilistic version of the algorithm.
- counter, a value used to determine when a location update has to be forwarded. It is incremented every time a packet is received. When its value becomes larger than *q*, the packet is forwarded, and *q* is subtracted from the counter. This property is only available for the deterministic version of the algorithm.

For the description of the algorithms, functions broadcast and receive have the following meaning:

- broadcast (id, position, time) sends the node ID, the position, and the timestamp associated with the position to all nodes at one hop.
- receive (& id, & position, & time) waits for the next broadcast message to be received, and returns the node ID, the position, and the timestamp from the content of the packet.

## 4. Performance Analysis

The aim of this section is to demonstrate the low complexity and scalability of our algorithm. In order to do so, we developed an analytical mathematical model and used it to determine the mean number of messages generated by each location update. We also show that the locally generated updates in the location tables by each location update were only related to the mean number of generated messages. Thus, we only focus on the mean number of messages generated per location update. The section is organized as follows. First, the cost of SFLS in terms of messages sent is estimated and this shows the scalability of the solution. Secondly, SFLS is compared with an optimized flooding algorithm used in the OLSR protocol.

### 4.1. Mathematical Model

Table 1 summarizes the notations used in this section. However, these notations are introduced in the text when they are needed in the description.

Let *N* be the number of nodes in the network, and $l_{i}$ be the length of the longest path from node *i* to any other node in the network. By definition, $l_{i}<N\;\forall i$.

Let $N_{i,h}$ be the number of nodes at *h* hops from node *i*. When there is no node at *h* hops, which is always possible with a nonzero probability, we set $N_{i,h}=0$), and by convention $N_{i,0}=1$.

Let $\bar{m_{i}}$ be the mean number of messages broadcast to update the location information of node *i* in the network. Node *i* is continuously broadcasting. Nodes at one hop from node *i* broadcast 1 in *q*. Nodes at two hops from node *i* broadcast 1 in $q^{2}$ (they receive 1 location update in *q* and forward them 1 in *q*), etc. As a result, $\bar{m_{i}}$ can be written:

(1) $$\begin{array}{ccc} \bar{m_{i}}\; & = & 1+\frac{1}{q}\;E\left(N_{i,1}\right)+\frac{1}{q^{2}}\;E\left(N_{i,2}\right)+...+\frac{1}{q^{l_{i}-1}}\;E\left(N_{i,l_{i}-1}\right)\\ \; & = & \;\sum_{j=0}^{l_{i}-1}\frac{E(N_{i,j})}{q^{j}} \end{array}$$

where E(N) is the expected value of *N*.

Let $\bar{u_{i}}$ be the mean number of location updates performed in the network every time node *i* broadcasts its new location information. Node *i* is continuously updating. Nodes at one hop are also continuously updating. Nodes at two hops update 1 in *q* (they receive only one update in *q* from nodes at one hop). Nodes at three hops update 1 in $q^{2}$, etc. As a result, $\bar{u_{i}}$ can be written as

(2) $$\begin{array}{ccc} \bar{u_{i}} & = & 1+N_{i,1}+\frac{1}{q}\;N_{i,2}+...+\frac{1}{q^{l_{i}-1}}\;N_{i,l_{i}}\\ & = & \left(q\sum_{j=1}^{l_{i}}\frac{N_{i,j}}{q^{j}}\right)+1\\ & = & q\times \bar{m_{i}}+1-q \end{array}$$

Consequently, $\bar{u_{i}}$ and $\bar{m_{i}}$ vary identically. In the following, we only focus on $\bar{m_{i}}$.

### 4.2. Node-Location Patterns

Equations (1) and (2) show that the mean number of both the sent messages and the updates for every new location update broadcast over the network depend on the number of nodes at each hop from node *i*.

In the following, the nodes were assumed to be uniformly distributed over the network according to a homogeneous Poisson point process of rate λ. The radio-transmission range of the nodes was assumed to be *R*.

Three different cases were considered: (1) cars located on a highway where all vehicles are on the same axis, moving in the same direction or (2) in the exact opposite direction, or (3) cars located in a city, and the movement of the other vehicles could be considered as a pseudorandom movement on a plane. To provide the complete picture, we also considered a 3D scenario. The most plausible scenario for this last case was not a VANET, but a set of drones flying together.

#### 4.2.1. One-Dimensional Scenario

For the highway scenario, nodes were assumed to be located on a rectangle of which the width was the width of the road, and the length was infinite. The width of the road was small compared to the broadcast range, and it was acceptable to consider that the nodes were on a line. In this case, our model was a line with node density λ, expressed in vehicles per meter. Since all nodes at distance *j* were in a segment, starting from the position of the farthest node at distance j−1 from *i*, nodes at distance *j* were in a segment of length *R*. Thus, the mean number of nodes at distance *j* is the mean number of nodes in a segment of length 2R (one segment to the right of *i* and one segment to the left of *i*).

**Figure 4:**
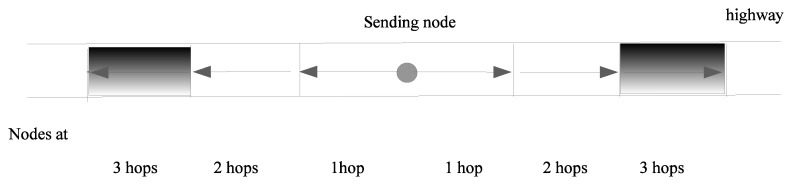
Node locations in 1D highway scenario.

Figure 4 shows the location of nodes at *n* hops according to the hypotheses above. For example, the grey rectangles, i.e., those at a distance between 2R and 3R from the center (i.e., node *i*), show the location of nodes at three hops.

The mean number of nodes at various distances from *i* is

(3) $$\begin{array}{ccc} E\left(N_{i,0}\right)=1\;\mathrm{and}\;E\left(N_{i,j}\right) & = & 2\lambda R\;\forall j>0. \end{array}$$

#### 4.2.2. Two-Dimensional Scenario

**Figure 5:**
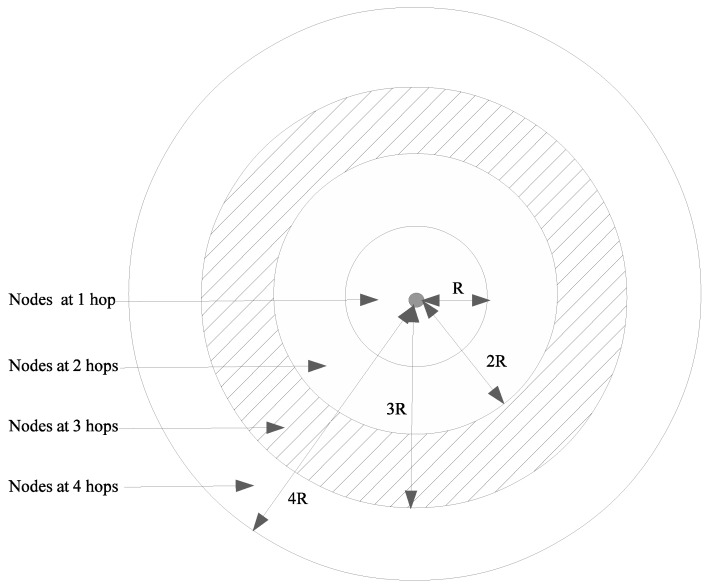
Node locations in a 2D urban scenario.

In this case, our model was a plane with node density λ, expressed in vehicles per square meter. The exact model would have to compute number of nodes $N_{i,j}$ at *j* hops from node *i*, and compute the mean value of $N_{i,j}$ averaged on the whole spatial configuration. Although this computation did not seem possible with state-of-the-art of stochastic geometry, we proposed to compute an upper bound. We assumed that the largest area in which $N_{i,j}$ nodes could be found was between the circle of radius (j−1)R and the circle of radius jR (see Figure 5). This occurred when density of nodes λ was very high. In this case, one could find nodes at j−1 hops exactly at distance (j−1)R from node *i*. However, this property has still not been rigorously demonstrated, but it was verified in the following simulations. The mean number of nodes at *j* hops from *i* was thus

$$E\left(N_{i,j}\right)\leq \lambda \pi R^{2}\left(j^{2}-{\left(j-1\right)}^{2}\right)=\lambda \pi R^{2}\left(2j-1\right)\;\forall j>0.$$

#### 4.2.3. Three-Dimensional Scenario

We developed the 3D case for the sake of completeness. However, these networks seem more suitable for a fleet of drones than for VANETs. The model is a 3D space of random nodes with density of nodes λ expressed in nodes per cubic meter. The exact model would require computing number $N_{i,j}$ of nodes at *j* hops from node *i*, and evaluating the mean value of $N_{i,j}$ averaged on many spatial configurations. As for the 2D case, the largest area in which the $N_{i,j}$ nodes could be located was assumed to be between the sphere of radius (j−1)R and the sphere of radius jR. This was obviously true when there was very high density of nodes λ, and this property was also assumed to hold with smaller densities. This was also verified by simulations.

The mean number of nodes at *j* hops from node *i* was

$$E\left(N_{i,j}\right)\leq \frac{4\lambda \pi R^{3}}{3}\left(j^{3}-{\left(j-1\right)}^{3}\right)=\frac{4\lambda \pi R^{3}}{3}\left(3j^{2}-3j-1\right)\;\forall j>0.$$

### 4.3. Algorithm Scalability

From the uniform distributions presented above, it was possible to determine the mean number of broadcast messages to update the location information of node *i* in a network.

We first considered a 1D network. The number of nodes at distance *j* from node *i* was computed above. Each of these nodes retransmitted 1 over $q^{j}$ times. Thus, the mean number of messages generated per location update is:

(4) $$\begin{array}{ccc} {\bar{m_{i}}}^{\left(1\right)} & = & 1+2\lambda R\sum_{j=1}^{l_{i}}\frac{1}{q^{j}} \end{array}$$

Since $\sum_{i=1}^{l_{i}}\frac{1}{q^{i}}=\frac{1}{q-1}\left(1-\frac{1}{q^{l_{i}}}\right)$, Equation (4) can be rewritten as

(5) $$\begin{array}{ccc} {\bar{m_{i}}}^{\left(1\right)} & = & 1+\frac{2\lambda R}{q-1}\left(1-\frac{1}{q^{l_{i}}}\right). \end{array}$$

For the probabilistic approach with the retransmission of a packet with probability *r*, we have

(6) $$\begin{array}{ccc} {\bar{m_{i}}}^{\left(1\right)} & = & 1+\frac{2\lambda Rr}{1-r}\left(1-r^{l_{i}}\right) \end{array}$$

We now consider a 2D network. The number of nodes at distance *j* from node *i* was upper-bounded above. Each of these nodes retransmitted 1 over $q^{j}$ times. The mean number of messages generated per location update could be upper-bounded as follows:

(7) $$\begin{array}{ccc} {\bar{m_{i}}}^{\left(2\right)} & \leq & 1+\lambda \pi R^{2}\;\sum_{j=1}^{l_{i}}\frac{2j-1}{q^{j}} \end{array}$$

Since

$$\sum_{i=1}^{l_{i}}\frac{\left(2i-1\right)}{q^{i}}=\frac{q+1}{{\left(q-1\right)}^{2}}+\frac{-\left(2l_{i}+1\right)q+2l_{i}-1}{q^{l_{i}}{\left(q-1\right)}^{2}},$$

Equation (7) can be rewritten as

(8) $$\begin{array}{c} {m_{i}}^{\left(2\right)}\leq 1+\lambda \pi R^{2}\left(\frac{q+1}{{\left(q-1\right)}^{2}}+\frac{-2\left(l_{i}+1\right)q+2l_{i}-1}{q^{l_{i}}{\left(q-1\right)}^{2}}\right). \end{array}$$

For the probabilistic approach, Equation (7) becomes

(9) $$\begin{array}{ccc} {m_{i}}^{\left(2\right)} & \leq & 1+\lambda \pi R^{2}\left(\frac{r(r+1)}{{\left(1-r\right)}^{2}}-\frac{r^{l_{i}}\left(\left(2l_{i}+1\right)r\right)-2l_{i}-1)}{{\left(1-r\right)}^{2}}\right). \end{array}$$

We now consider a 3D network. The number of nodes at distance *j* from node *i* was upper-bounded above. Each of these nodes retransmitted 1 over $q^{j}$ times. The mean number of messages generated per location update could be upper-bounded as follows:

(10) $$\begin{array}{ccc} {\bar{m_{i}}}^{\left(3\right)} & \leq & 1+\lambda \frac{4}{3}\pi R^{3}\;\sum_{j=1}^{l_{i}}\frac{3j^{2}-3j-1}{q^{j}} \end{array}$$

Since

(11) $$\begin{array}{ccc} \sum_{j=1}^{l_{i}}\frac{3j^{2}-3j-1}{q^{j}} & = & \frac{-3\left(l_{i}^{2}+l_{i}+1\right)q^{2}}{{\left(q-1\right)}^{3}q^{l_{i}}}+\frac{\left(6l_{i}^{2}-4\right)q-3l_{i}^{2}-6l_{i}-10}{{\left(q-1\right)}^{3}q^{l_{i}}}\\ \; & \; & +\frac{q^{2}+4q+1}{{\left(q-1\right)}^{3}}, \end{array}$$

Equation (10) can be rewritten as

(12) $$\begin{array}{ccc} {\bar{m_{i}}}^{\left(3\right)} & \leq & 1+\lambda \frac{4}{3}\pi R^{3}(\frac{\left(-3l_{i}^{2}+3l_{i}-1\right)q^{2}}{{\left(q-1\right)}^{3}q^{l_{i}-1}}\\ \; & \; & +\frac{\left(6l_{i}^{2}-12l_{i}+2\right)q-3l_{i}^{2}-7}{{\left(q-1\right)}^{3}q^{l_{i}-1}}+\frac{q^{2}+4q+1}{{\left(q-1\right)}^{3}}). \end{array}$$

For the probabilistic approach, Equation (10) becomes

(13) $$\begin{array}{ccc} {\bar{m_{i}}}^{\left(3\right)} & \leq & 1+\lambda \frac{4}{3}\pi R^{3}(\frac{\left(-3l_{i}^{2}+9l_{i}-7\right)r^{2}}{r^{l_{i}-1}{\left(1-r\right)}^{3}}\\ \; & \; & +\frac{\left(6l_{i}^{2}-12l_{i}+2\right)r-3l_{i}^{2}+3l_{i}-1}{r^{l_{i}-1}{\left(1-r\right)}^{3}}+\frac{r(r^{2}+4r+1)}{{\left(1-r\right)}^{3}}). \end{array}$$

From Equations (5), (8), and (12), it is straightforward to demonstrate that

(14) $$\begin{array}{ccc} \lim_{l_{i}\to +\infty }{\bar{m_{i}}}^{\left(1\right)} & = & 1+\frac{2\lambda R}{q-1}. \end{array}$$

(15) $$\begin{array}{c} \lim_{l_{i}\to +\infty }{\bar{m_{i}}}^{\left(2\right)}\leq 1+\lambda \pi R^{2}\left(\frac{q+1}{{\left(q-1\right)}^{2}}\right) \end{array}$$

and

(16) $$\begin{array}{c} \lim_{l_{i}\to +\infty }{\bar{m_{i}}}^{\left(3\right)}\leq 1+\lambda \frac{4}{3}\pi R^{3}\left(\frac{q^{2}+4q+1}{{\left(q-1\right)}^{3}}\right). \end{array}$$

These limits are of the 1+a×M form, where *M* is the mean number of neighbors of node *i*, and *a* only depends on *q*. One can easily derive the mean number of transmissions for node *i* with a simple flooding:

(17) $$\begin{array}{c} {\bar{M_{i}}}^{\left(1\right)}=2\lambda Rl_{i}≃N \end{array}$$

(18) $$\begin{array}{c} {\bar{M_{i}}}^{\left(2\right)}=\pi \lambda R^{2}l_{i}^{2}≃N \end{array}$$

(19) $$\begin{array}{c} {\bar{M_{i}}}^{\left(3\right)}=\frac{4}{3}\pi \lambda R^{3}l_{i}^{3}≃N. \end{array}$$

The comparison between 1+a×M and *N* clearly shows the advantage and scaling properties of our proposed algorithm.

### 4.4. Numerical Results

In our numerical examples, densities λ of the nodes were chosen so that the mean number of neighbors of a node was 20; transmission range was R=100.

**Figure 6:**
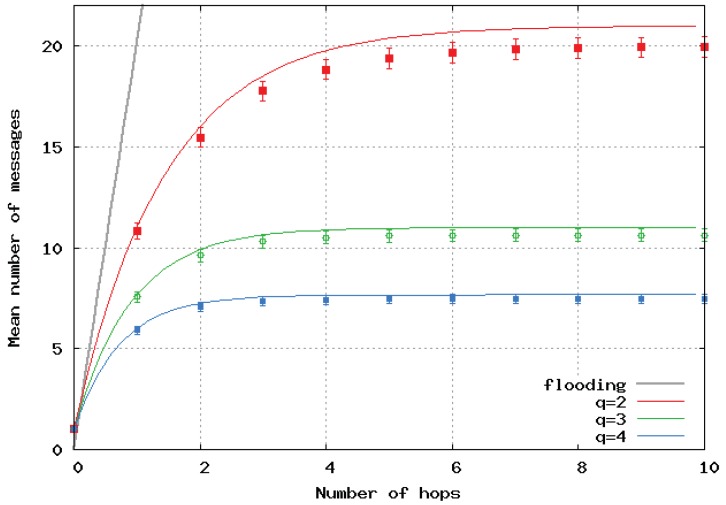
Mean number of messages sent per location update with respect to number of hops in 1D homogeneous network.

Figure 6 presents the mean number of broadcast messages per location update versus the maximal path lengths computed by the analytical model and simulations in the case of a homogeneous linear (1D) network. Analytical results are represented by a continuous line, and simulation results by dots and error bars. The same color is used for the same value of *q* for the simulations and the analytical model. We observed very good matching of the two approaches. The black curve shows the number of messages with the pure flooding algorithm, and it clearly shows the great benefit of SFLS.

**Figure 7:**
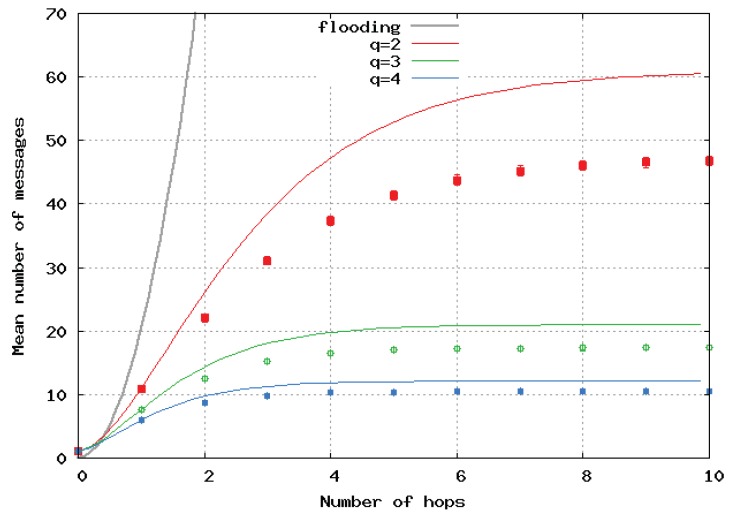
Mean number of messages sent per location update with respect to number of hops in 2D homogeneous network.

**Figure 8:**
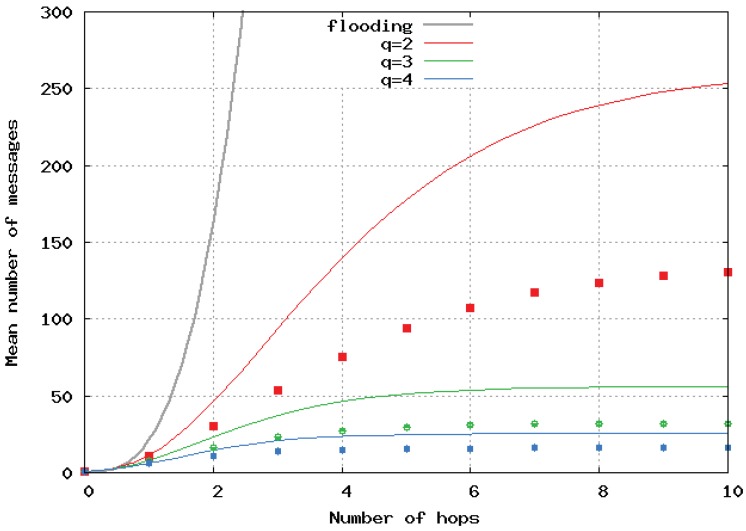
Mean number of messages sent sent per location update with respect to number of hops in 3D homogeneous network.

Figure 7 and Figure 8 show the mean number of messages per location update versus the path length computed by the simulation and its upper-bound, analytically computed for homogeneous 2D and 3D networks, respectively. The analytical results are represented by a continuous line, and the simulation results by dots and error bars. Simulations presented in Figure 6, Figure 7 and Figure 8 show error bars corresponding to a confidence level of 95%. The upper bounds largely overestimated the number of broadcast messages in 2D and 3D networks. This is because these bounds were only tight when the density of nodes was very high. When the number of hops $l_{i}$ increased, it can be seen that increasing *q* strongly decreased the mean number of generated messages. The benefit of having a large *q* is greater for 2D and even greater for 3D networks. Here, again, we observe the great benefit of the algorithm we propose over pure flooding.

### 4.5. Comparison with Other Flooding Techniques

The section above shows that SFLS scaled well with the total number of nodes in the network since the mean number of retransmissions was of form 1+a×M, where *M* is the mean number of neighbors of a node (M is constant since the network is homogeneous.). However, if a proactive routing protocol is already implemented in the network, one may consider using information that is already present to help the flooding of location information. The MPR flooding of the optimized-link-state -routing (OLSR) protocol, which exploits the information included in the Hello packets, was shown to be very efficient and, in particular, more efficient than the dominating set flooding (see [8]). We propose to disseminate location information with the multipoint relays (MPRs) of OLSR. The main idea is to ensure the dissemination of a packet using special nodes called multipoint relays (MPRs). The multipoint-relay set of a node is a minimal set of its neighbors that can reach all of its two-hop neighbors (The two-hop neighbors of a node are the neighbors of the neighbors that are not one-hop neighbors. Using MPRs recursively is enough to broadcast a packet to all network nodes. The MPR set is minimal in terms of number of nodes.).

We now consider a 2D homogeneous network. Using MPR flooding, an upper bound on the total number of retransmissions required to complete full flooding is $N/M^{\frac{2}{3}}$, where *M* denotes the mean number of neighbors of a node. Thus, the upper bound of the total number of retransmissions for MPR flooding is

$$\frac{N}{M^{\frac{2}{3}}}=\frac{\lambda \pi R^{2}l_{i}^{2}}{M^{\frac{2}{3}}}=M^{\frac{1}{3}}l_{i}^{2}.$$

For SFLS, where flooding is based on the retransmission of 1 in *q* messages, the upper bound on the total number of retransmissions per update is

$$1+\lambda \pi R^{2}\left(\frac{q+1}{{\left(q-1\right)}^{2}}+\frac{\left(1-2l_{i}\right)q+2l_{i}-3}{{\left(q-1\right)}^{l_{i}-1}{\left(q-1\right)}^{2}}\right),$$

which can be written as

$$1+M\left(\frac{q+1}{{\left(q-1\right)}^{2}}+\frac{\left(1-2l_{i}\right)q+2l_{i}-3}{{\left(q-1\right)}^{l_{i}-1}{\left(q-1\right)}^{2}}\right).$$

**Figure 9:**
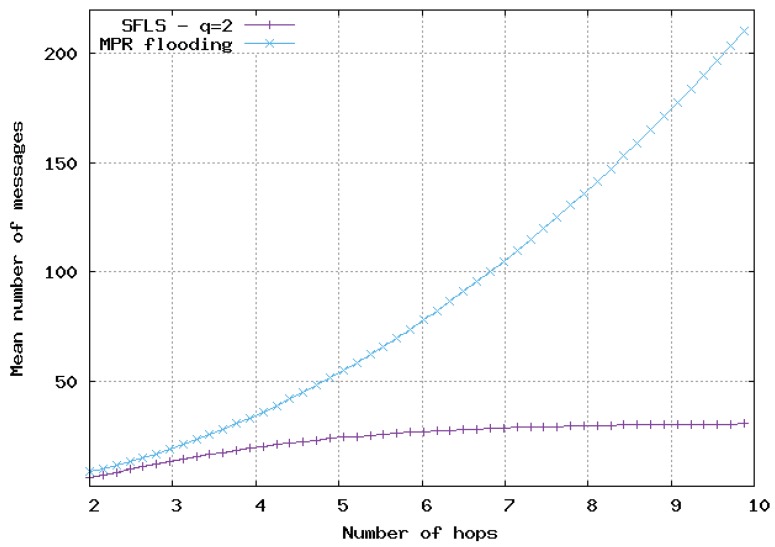
Mean number of messages sent for update with respect to number of hops in 2D homogeneous network, M=10. Comparison of SFLS with the MPR flooding.

**Figure 10:**
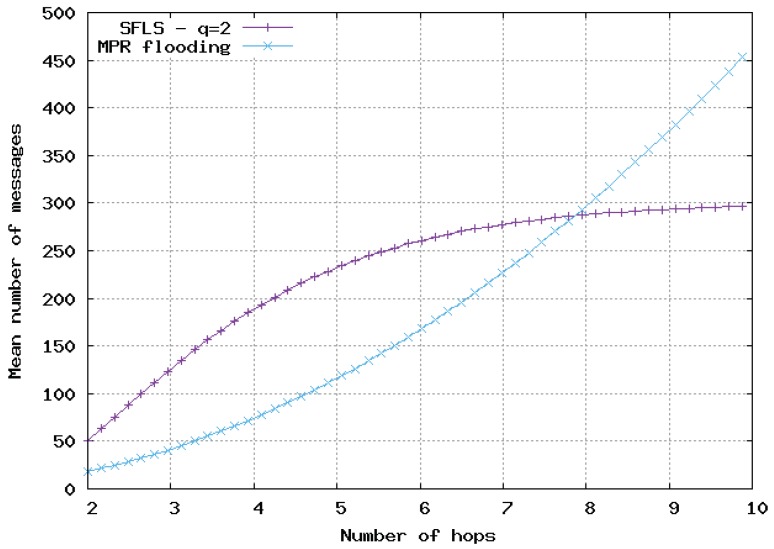
Mean number of messages sent for update with respect to number of hops in 2D homogeneous network, M=100. Comparison of SFLS with the MPR flooding.

**Figure 11:**
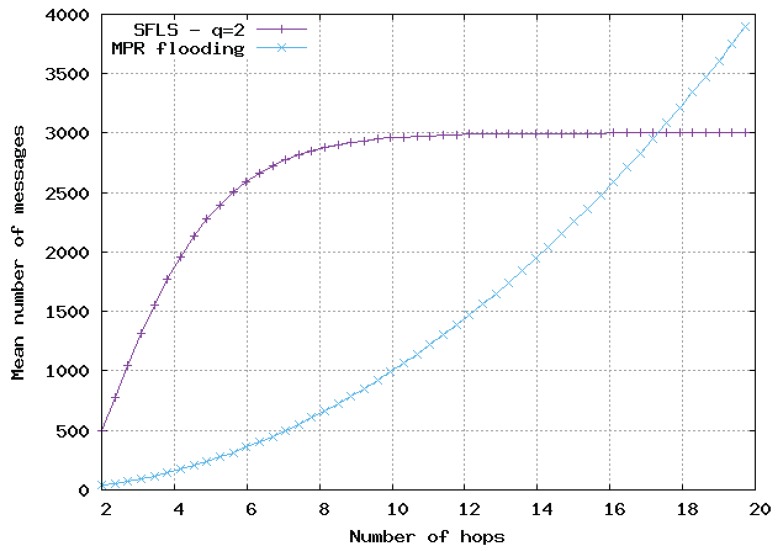
Mean number of messages sent for update with respect to number of hops in 2D homogeneous network, M=1000. Comparison of SFLS with MPR flooding.

In Figure 9, Figure 10 and Figure 11, the flooding overhead of SFLS is compared with the overhead of OLSR in homogeneous 2D networks. We varied density λ or equivalently *M*, i.e., the mean number of neighbors for a random node. In Figure 9, Figure 10 and Figure 11, *M* is equal to 10, 100 and 1000, respectively.

For SFLS, the developed upper bound in the analytical model was used. Our protocol always outperformed OLSR when the number of hops was large. However, OLSR outperformed our scheme for a small number of hops or when the network was very dense.

As a result, unlike other flooding-based location services, the semiflooding location service is scalable in terms of the number of messages that must be sent to keep all the nodes updated, the number of updates that are performed in the network, and the time required to answer a location request.

## 5. Conclusions and Future Work

SFLS, the semiflooding location service, an efficient and yet simple solution for the dissemination of location information in VANETs, was described in this paper. We analyzed the performance of this protocol with a uniform Poisson point process of nodes in 1D, 2D, and 3D networks. Even though the basis of this protocol is a broadcast scheme, we showed that the number of messages per update remains very low. For different cases, we built an analytical model to upper bound the number of messages per update, and carried out simulations to verify our computations. The matching of the analytical model and the simulations was very good. We also compared SFLS with the broadcast optimization scheme of OLSR: the multipoint-relay technique. We presented two case studies where SFLS and the multipoint-relay technique performed differently. In dense or very dense networks, the MPR scheme was better, but SLFS outperformed the MPR flooding of OSLR in sparse or moderately dense networks, and in any case, when the number of hops was large.

It would be interesting to combine SFLS with a location-prediction technique based on node kinematics to refine their positions between the reception of two consecutive SFLS updates. This idea could be the starting point for further studies.

## Figures and Tables

**Table 1:** Notations used in analytical model.

Number of network nodes	*N*
Density of network nodes	λ
Transmission range	*R*
Mean number of neighbors for a node	*M*
Longest past from node *i* to any other node in the network	$l_{i}$
One over *q* messages is repeated in the deterministic algorithm	*q*
re-transmission probability in the probabilistic scheme	*r*
Number of nodes at *h* hops from node *i*	$N_{i,h}$
Mean number of messages and mean number of updates	$\bar{m_{i}},\bar{u_{i}}$
Number of messages sent by node *i* in kD case with our algorithm	${\bar{m_{i}}}^{\left(k\right)}$
Number of messages sent by node *i* in kD case with flooding	${\bar{M_{i}}}^{\left(k\right)}$
